# An Update on the Management of Endodontic Biofilms Using Root Canal Irrigants and Medicaments

**Published:** 2014-03-08

**Authors:** Zahed Mohammadi, Mohammad Karim Soltani, Sousan Shalavi

**Affiliations:** a *Iranian Center for Endodontic Research (ICER), Research Institute of Dental Sciences, Shahid Beheshti University of Medical Sciences, Tehran, Iran and Iranian National Elite Foundation, Tehran, Iran; *; b *Department of Orthodontics, Hamedan University of Medical Sciences, Hamedan, Iran;*; c * Private Dental Practice, Hamedan, Iran*

**Keywords:** Biofilms, Calcium Hydroxide, Chlorhexidine, Intracanal Irrigants, Lasers, MTAD, Ozone, Root Canal, Periapical Lesions, Sodium Hypochlorite

## Abstract

Microbial biofilm is defined as a sessile multicellular microbial community characterized by cells that are firmly attached to a surface and enmeshed in a self-produced matrix of extracellular polymeric substances. Biofilms play a very important role in pulp and periradicular pathosis. The aim of this article was to review the role of endodontic biofilms and the effects of root canal irrigants, medicaments as well as lasers on biofilms A Medline search was performed on the English articles published from 1982 to 2013 and was limited to papers published in English. The searched keywords were “Biofilms AND endodontics”, “Biofilms AND sodium hypochlorite”, "Biofilms AND chlorhexidine", "Biofilms AND MTAD", "Biofilms AND calcium hydroxide", “Biofilms AND ozone”, “Biofilms AND lasers” and "Biofilms AND nanoparticles". The reference list of each article was manually searched to find other suitable sources of information.

## Introduction

Many microorganisms are able to form surface-attached microbial communities, known as biofilms. In fact, biofilms are communities of microorganisms attached to a surface and embedded in a matrix of polysaccharides and proteins forming a slimy layer [1]. The matrix typically takes 85% of the volume of a biofilm [1]. Oral bacteria have the capacity to form biofilms on distinct surfaces ranging from hard to soft tissues. The characteristics of the biofilms depend upon the residing bacterial species, composition and structure of the surface or substratum, and the conditioning layer coating the surfaces on which they are formed. Water constitutes 80% of the oral biofilms, while the organic and inorganic fractions form approximately 20% of the biofilm structure [2].

**Definition of biofilm**

Biofilm is a mode of bacterial growth in which dynamic communities of interacting sessile cells are irreversibly attached to a solid surface, as well as each other, and are embedded in a self-made matrix of extracellular polymeric substances. A microbial biofilm is considered as a community that meets the following criteria: it must possess the abilities to self-organize (autopoiesis), resist environmental perturbations (homeostasis), must be more effective in association than in isolation (synergy), and respond to environmental changes as a unit rather than single individuals (communality) [3].

**Adaptation mechanisms**

Microorganisms undergo a wide range of physiological and morphological adaptations in response to environmental changes. In biofilms, different gradients of chemicals, nutrients, and oxygen create variable micro-environments to which micro-organisms must adapt to survive. The perception and processing of chemical information from the environment, forms a central part of the regulatory control over these adaptive responses. Adaptation to a biofilm life-style involves regulation of a vast set of genes, and the microorganisms are thus able to optimize phenotypic properties for the particular environment. Consequently, biofilm microorganisms differ phenotypically from their planktonic counterparts [4]. Formation of biofilms is a stepwise process (Figure 1). Although the structural organization of biofilms and the composition/activities of the colonizing microorganisms in various environments may be different, the establishment of a micro-community on a surface seems to follow essentially the same series of developmental stages, including deposition of a conditioning film, adhesion and colonization of planktonic microorganisms in a polymeric matrix, co-adhesion of other organisms, and detachment and releasing of biofilm microorganisms into their surroundings [5]. Evidence is emerging that expression of genes required during the various stages is well-regulated [6-8]. 

The coordinated gene expression is regulated through various signal transduction systems that induce cascades of reactions, which leads to the induction or inhibition of gene transcription. In some cases the external stimulus is inherent in the environment, although the molecules involved are mostly unknown. For other systems, the stimulus represents known molecules. The so-called two-component regulatory systems are frequently involved in the control of gene expression in response to various stimuli. 

Two-component regulatory systems include a histidine kinase and a response regulator. Several such two-component systems exist in gram-positive and gram-negative microorganisms [9-11]. They play important roles in signal transduction and may be essential sensors for adaptation to a biofilm life [3, 12]. The external stimulus is sensed by the transmembrane histidine kinase receptor that then catalyzes an intracellular ATP-dependent autophosphorylation [12]. The phosphoryl group is subsequently transferred to a conserved aspartate residue of the regulatory domain of its cognate response regulator. In the most sophisticated systems, activation of the response regulator occurs through multistep phosphorelay cascades. The phosphorylated form of the response regulator will influence transcription by binding to the promoter sequences of genes under its control, resulting in gene activation or repression [3, 12].

**Types of endodontic biofilms**

In endodontics, biofilms can be divided into intracanal, external root (cementum), and periapical biofilms [2].

***Intracanal biofilms***

For the first time, by using transmission electron microscopy (TEM), Nair examined the root canal contents of 31 teeth, which had gross coronal caries and the periapical inflammatory tissue was attached to root surface upon extraction; besides the microstructure of the inflammatory tissues, the major bulk of the organisms existing as loose collections of cocci, rods, filaments and spirochetes was observed as well [13]. In a scanning electron microscopy (SEM) study, Sen *et al.* showed that the bacteria formed dense colonies on the canal walls as well as in inter/intra tubular dentin. Furthermore, they observed fungi capable of forming dense, but separate colonies all over the root canal walls [14].

It has been shown that when *E. faecalis* was grown under aerobic nutrient-rich condition, it produced irregularly shaped amorphous macro-structures of 500-1000 µm in dimension [15]. According to George *et al*., these biofilms showed an increased elemental concentration of Ca and P but the Ca/P ratio was similar to that of dentin*.* [15]. When examined by SEM and confocal laser scanning microscope (CLSM), *E. faecalis* specimens kept under nutrient-rich, anaerobic conditions showed mature biofilms with apparent water channels on the root canal wall. Bacterial cells suspended within the biofilm structure were evident from the CLSM observations. 

The examination of biofilms formed under nutrient-deprived, aerobic environment showed obvious signs of surface degradation of dentin. A study revealed that pure cultures of *E. faecalis* inoculated to calcium hydroxide-medicated or non-medicated root canals were able to form a biofilm structure on canal walls [16]. Another study demonstrated that even after instrumentation, irrigation and obturation in a one-visit treatment, microorganisms existed as biofilms in untouched locations in the main canal, isthmi and accessory canals in 14 out of the 16 endodontically-treated teeth [17].

Using SEM, light microscopy, and CLSM, Kishen *et al.* demonstrated different stages in the interaction of *E. faecalis* with root canal dentin [18]. Furthermore, the re-precipitation of a bacterial-induced apatite on mature biofilm was also observed. This ability of *E. faecalis* to form such calcified biofilm on root canal dentin may be a factor that contributes to its persistence.

***External root-surface (cementum) biofilms***

According to Ingle *et al.*, cementum biofilms have been reported in teeth with asymptomatic apical periodontitis as well as teeth with chronic apical abscesses and a sinus tract [2]. Using SEM, Tronstad *et al.* showed that the apex of the roots adjacent to the apical foramen was coated with a continuous, smooth, structure-less layer containing a variety of bacterial forms [19]. The organisms were identified as cocci and rods with presence of fibrillar forms, to a lesser degree. The presence of calculus-like deposits on the root tip of teeth with secondary (post-treatment) apical periodontitis has been revealed [20]. Calcified biofilms on the apical root surface of teeth with lesions refractory to conventional root canal treatment has also been demonstrated [21].

Siqueira and Lopes used SEM to assess the extracted teeth with extensive caries and asymptomatic periradicular lesions [22]. It was observed that cocci and rods were restricted to the root canal and in only one tooth; bacteria were seen beyond the apical foramen. Most bacteria appeared suspended in the fluid phase of the root canal. It was remarked that the presence of bacteria at or outside the apical foramen might not necessarily be a true condition, but rather a function of extrusion of bacterial colonies during tooth extraction. Based on their findings, extraradicular infection in terms of root tip aggregations may not be a common occurrence in untreated teeth with infected pulps.

Using SEM, Lomcali *et al.* assessed the apical root surfaces of teeth with chronic apical periodontitis [23]. In addition to lacunar resorption sites and the clastic cells over their surfaces, presence of bacteria and fungi in some of the lacunae and periapical bacterial plaque was observed around the main apical foramen.

In another study, Leonardo *et al.* found that the presence of chronic periapical lesions caused severe changes in the apical structure with destruction of fibers and different degrees of forming cementum resorption lacunae in which bacterial biofilm persisted [24]. Rocha *et al.* showed similar findings in primary teeth [25]. Noiri *et al.* analyzed the presence of biofilms on root tips of extracted teeth with refractory periapical pathosis and the removed gutta-percha points during endodontic treatment by SEM [26]. Gutta-percha points sticking out through the apex were almost completely covered with glycocalyx-like structures. Bacteria, mostly filaments or long rods, were seen on the external root surfaces of the extracted teeth.

***Periapical biofilms***

According to Ingle *et al.*, these types of biofilms may or may not be dependent on the root canal infection [2]. Members of the genus *Actinomyces* and *Propionibacterium propionicum* have been demonstrated in asymptomatic periapical lesions refractory to endodontic treatment [27]. This condition consists of establishment of microorganisms in the periapical tissues, either by their adherence to the apical root surface in the form of biofilm-like structures [19] or within the body of the inflammatory lesion, usually as cohesive colonies [28].

Although the exact pathogenicity mechanism of *Actinomyces* species has not been completely clarified, there is some evidence that may help with explaining infections caused by these microorganisms. Possessing fimbrial structures may play a role in bacterial coaggregation within the root canal and can be important for bacterial survival in the ecosystem. In addition, fimbriae would enable *Actinomyces* cells to adhere to the root canal wall and to dentinal debris forced out through the apical foramen during treatment, and to cling to other bacteria or host cells as they advance into the periapical tissues [29].

*Actinomyces* species usually have a hydrophobic cell-surface, which facilitates their uptake (phagosytosis) by leukocytes. Figdor and Davies suggested that both the fimbriae-like structures and the matrix of the outer coat surrounding the bacteria can help the cells to aggregate into cohesive colonies of tangled filaments [30]. Moreover, strains associated with post-treatment disease, grow as intertwining filaments that form granulae within the host tissues [29, 31]. Actinomycotic colonies may live in an equilibrium with host tissues that enables them to maintain a chronic periapical inflammation without necessarily inducing an acute response. Very high numbers of *Actinomyces* cells are usually needed to form persistent infections [32]. The low pathogenicity of these microorganisms and the consequent minimal host response may be the reasons for the perpetuation of the chronic periapical lesion.

**Mechanisms of antimicrobial resistance of biofilm**

There are several mechanisms for biofilms to resist antimicrobial agents. By encouraging growth of species beneficial to the biofilm, quorum sensing (communication with one another) can influence the structure of the biofilm [5]. Biofilm bacteria live in a low metabolic state, a slower growth-rate and production of exopolysaccharides [33]. Subpopulations of bacteria in a biofilm form a phenotypic state (altered gene expression) where they are highly protected [1].

The polysaccharide matrix of biofilms can retard diffusion of antibiotics. Furthermore, extracellular enzymes such as β-lactamase may become trapped and concentrated in the matrix that ends up in inactivation of β-lactam antibiotics [34]. Bacteria protect themselves by being located within the interior part of a biofilm. Hence medicaments will only act on the micro-organisms in the peripheral portion of the biofilm. Bacterial cells residing within a biofilm grow more slowly than planktonic cells and as a result, antimicrobial agents act more slowly [34]. Depletion of nutrients or accumulation of waste products can result in bacteria entering a non-growing state which protects them from the antibiotics [1], as well as the dose and frequency of exposure to the antimicrobial agent [35]. Chemical changes in the biofilm to face the environment, where the lack of oxygen inhibits some antibiotics and also the accumulated acidic waste, leads to a change in pH which has an antagonizing effect on the antibiotic [36].

**Effects of endodontic irrigants and medicaments on biofilms**

Antimicrobial agents have often been developed and optimized for their activity against fast growing, dispersed populations containing a single microorganism [5, 37]. However, microbial communities in biofilms are remarkably difficult to eradicate with antimicrobial agents and microorganisms in mature biofilms can be notoriously resistant for reasons that have yet to be adequately explained. There are reports showing that microorganisms grown in biofilms could be 1000-1500 times more resistant to antimicrobial agents that planktonic bacteria [5].

***Sodium hypochlorite***

Spratt *et al.* showed that sodium hypochlorite (NaOCl) was the most effective anti-microbial irrigant followed by the iodine solution [35]. Clegg *et al.* indicated that 6% NaOCl was the only irrigant capable of both rendering bacteria nonviable and physically removing the biofilm [38].

Ozok *et al.* compared growth and susceptibility to different concentrations of NaOCl of mono- and dual-species biofilms of *Fusobacterium nucleatum* or *Peptostreptococcus* *micros* at 24 or 96 h,* in vitro* [38]. Results revealed that although at 24 h the dual-species biofilms had similar viable counts to those of monospecies, they were more resistant to NaOCl. At 96 h, both microorganisms had higher viable counts and were more resistant to NaOCl in dual-species biofilms than in monospecies. Mixed-species biofilms of *F. nucleatum* and *P. micros* showed a time-dependent synergy in growth and resistance to NaOCl. Using a flow cell system, Dunavant *et al.* showed that the percentage of killed bacteria in biofilms after using 6% and 1% NaOCl was above 99.99% and 99.78%, respectively [34]. Giardino *et al.* evaluated the efficacy of 5.25% NaOCl and MTAD against *E. faecalis* biofilm and found that only 5.25% NaOCl can disgregate and remove the biofilm at every time [39]. Williamson *et al.* indicated that 6% NaOCl (Chlor-Xtra, Vista Dental, Racine, Mi, USA) was significantly superior against *E. faecalis* biofilms compared to 2% chlorhexidine (CHX) and CHX-Plus [40]. Arias-Moliz *et al.* revealed that NaOCl was the most effective agent, capable of eradicating the biofilms after 1 min with a concentration of 0.00625% [41]. Chavez de Paz *et al.* tested the *in situ* effect of antimicrobials and Alkali (PBS adjusted to pH=12 with NaOH) on biofilms of *Enterococcus faecalis*, *Lactobacillus paracasei*, *Streptococcus anginosus*, and *Streptococcus gordonii* isolated from root canals with persistent infections [42]. Findings indicated that 1% NaOCl affected the membrane integrity of all organisms and removed most biofilm cells. According to Prabhakar *et al.*, 5% NaOCl showed maximum antibacterial activity against *E. Faecalis* biofilm formed on tooth substrate [43]. Bhuva *et al.* demonstrated that both conventional syringe irrigation and passive ultrasonic irrigation with 1% NaOCl were effective in complete removal of intraradicular *E. faecalis* biofilms [44].

According to Liu *et al.,* biofilms of starved cells of *E. faecalis* were more resistant to 5.25% NaOCl than stationary cells and the impact of 5.25% NaClO on them decreased as the biofilm matured [45]. Ozdemir *et al.* revealed that the combined application of EDTA and NaOCl significantly reduces the amount of intracanal biofilm [46].

Soares *et al.* demonstrated that the irrigation regimen based on the alternating use of NaOCl and EDTA seems to be a promising endodontic tool because it promoted the elimination of root canal *E. faecalis* biofilms [47]. Del Carpio-Perochena showed that in comparison with the 5- and 15-min contact times, a 30-min application of NaOCl is necessary to have higher values of biofilm dissolution and to increase the cleaning of the dentin independent of the solution concentration [48]. It has been revealed that 1% NaOCl was the only irrigant that had a significant effect on biofilm viability and architecture [49]. Seet *et al.* indicated that syringe irrigation and sonic activation with NaOCl showed reduced numbers of bacterial cells on the radicular dentine but were not effective in eliminating *E. faecalis* in the dentinal tubules [50]. Laser activation of NaOCl resulted in clean dentine walls and undetectable levels of bacteria within dentinal tubules. According to Neelakantan *et al.*, 3% NaOCl showed maximum antibacterial activity against *E. faecalis* bioﬁlm formed on the tooth substrate [51].

***Chlorhexidine***

Clegg *et al.* indicated that 2% CHX, was not capable of disrupting biofilms [52]. Dunavant *et al.* evaluated the efficacy of 2% CHX against *E. faecalis* biofilms after 1 or 5 min [34]. Findings showed that there was no significant relationship between time and percentage of killed microorganisms which was 60.49%. On the other hand, a study by Lima *et al.* demonstrated that 2% CHX-containing medications were able to thoroughly eliminate most of the 1- and 3-day biofilms of *E. faecalis* [53]. Williamson *et al.* indicated that 2% CHX was significantly less effective against *E. faecalis* biofilm compared to 6% NaOCl [40]. Arias-Moliz *et al.* showed that CHX eradicated biofilm after 5 min at 2% concentration [54]. According to Shen *et al.* CHX-Plus (2% CHX with surface modifiers, Vista Dental, Racine, Mi, USA) showed higher levels of bactericidal activity at all exposure times than 2% CHX [55]. Chavez de Paz *et al.* showed that 2.5% CHX had a mild effect on the membrane integrity of *E. faecalis* and removed only 50% of its biofilm cells [42]. Arias-Moliz *et al.* indicated that the association of 0.1% and 0.05% cetrimide with any concentration of CHX, whether in combined or alternating application, effectively eradicated *E. faecalis* biofilms at all tested contact times [41]. Shen *et al.* showed that the combined use of CHX with mechanical agitation had a more pronounced antimicrobial effect against the biofilms [55]. It has been revealed that bacteria in mature biofilms and nutrient-limited biofilms are more resistant to CHX than in young biofilms.

Del Carpio-Perochena *et al.* showed that 2% CHX did not dissolve the biofilms [48]. Du *et al.* demonstrated that 5 min treatment with Atmospheric Pressure Nonequilibrium Plasmas (APNPs) or 2% CHX, killed the majority of bacteria in *E. faecalis* biofilms [56]. It has been shown that treating *E. faecalis* biofilms with dextranase or DNase I, effectively sensitized the biofilms to 2% CHX [57]. Using an intraorally infected dentin biofilm model, Ordinola-Zapata *et al.* showed that 2% CHX gel was more effective than calcium hydroxide against *E. faecalis* biofilms [49].

***MTAD (a mixture of a tetracycline isomer, an acid, and a detergent)***

Dunavant *et al.* showed that Biopure MTAD (Dentsply, Tulsa Dental, Tulsa, OK, USA), which contains 150 mg/5 mL concentration of doxycycline, killed 16.08% of bacterial cells in *E. faecalis* biofilms [34]. Giardino *et al.* showed that MTAD was not able to disintegrate and remove bacterial biofilms [40]. Prabhakar *et al.* showed that MTAD was not able to remove *E. faecalis* biofilm [43]. Pappen *et al.* revealed that MTAD was less effective than Tetraclean (containing 50 mg/5 mL concentration of doxycycline, Ogna Laboratori, Farmaceutici, Milano, Italy) against *E. faecalis* biofilm *in vitro *[58]. Stojicic *et al.* showed that MTAD were unable to kill all plaque bacteria in 30 sec, and some *E. faecalis* cells survived even after 3 min of exposure [59]. Tong *et al.* indicated that adding nisin to MTAD enhanced its effectiveness against *E. faecalis* biofilm [60].

***Iodine compounds***

Spratt *et al.* investigated the effectiveness of some root canal irrigants against single-species biofilms of *Prevotella* *intermedia*, *Peptostreptococcus* *micros*, *Streptococcus intermedius, Fusobacterium nucleatum *and* Enterococcus faecalis* [35]*.* Findings revealed that iodine and NaOCl were more effective than CHX except against *P. micros *and* P. intermedia *for which they were all 100% effective. Iodine and NaOCl elicited a 100% kill bacteria elimination after 1 h incubation for all used strains. However, after 15 min, they showed differing bactericidal effects depending on the strain. Abdullah *et al.* revealed that 10% povidone iodine was less effective against *E. faecalis* biofilm compared to NaOCl [61].

***Calcium hydroxide***

Using SEM and SCLM, Distel *et al.* reported that despite intracanal dressing with calcium hydroxide (CH), *E. faecalis* formed biofilms in root canals [16]. In another study, Chai *et al.* reported that CH was 100% effective in eliminating *E. faecalis* biofilm [62]. Brandle *et al.* investigated the effects of growth condition (planktonic, mono- and multi-species biofilms) on the susceptibility of *E. faecalis*, *Streptococcus sobrinus, Candida albicans, Actinomyces naeslundii *and* Fusobacterium nucleatum* to alkaline stress [63]. Findings demonstrated that planktonic microorganisms were most susceptible; only *E. faecalis* and *C. albicans* survived in saturated solution for 10 min, the latter also survived for 100 min. Dentine adhesion was the major factor in improving the resistance of *E. faecalis* and *A. naeslundii* to CH, whereas the multispecies context in a biofilm was the major factor in promoting resistance of *S. sobrinus* to the disinfectant. In contrast, the *C. albicans* response to CH was not influenced by growth conditions.

In summary, few studies have been conducted on the antimicrobial potential of CH on biofilms and they have demonstrated inconsistent results. Further studies are required to elucidate the anti-biofilm efficacy of CH.

**Advanced agents**

***Nanoparticles***

Nanoparticles are microscopic particles with one or more particle dimensions in the range of 1–100 nm. Nanoparticles are recognized to have properties that are very unique compared to their bulk or powder counterparts [64]. In root canal therapy, nanoparticles may be applied as slurry or in combination with sealers. They have the ability to diffuse antimicrobial components deep in dentin tissue. The successful application of nanoparticles in endodontics will depend on both the effectiveness of antimicrobial nanoparticles and the delivery method used to disperse these particles into the anatomical complexities of the root canal system.

Magnesium oxide (MgO) and calcium oxide (CaO) slurries acted on both gram-positive and gram-negative bacteria in a bactericidal manner [38], while Yamamoto showed that zinc oxide (ZnO) slurry acted in a bacteriostatic manner and exhibited stronger antibacterial activity against gram-positive than gram-negative bacteria [65]. Sawai *et al.* showed that by generating active oxygen species, such as hydrogen peroxide and superoxide anion radical antibacterial agents, powders of MgO, CaO, and ZnO exert their antibacterial effect [66]. The electrostatic interaction between positively charged nanoparticles and negatively charged bacterial cells, and the accumulation of a large number of nanoparticles on the bacterial cell membrane, have been associated with the increase in membrane permeability and rapid loss of membrane function [67].

Feng *et al*. showed that silver ions inactivate proteins and inhibit the ability of DNA to replicate [68]. According to Kim *et al.* nanoparticles synthesized from powders of silver (Ag), copper oxide (CuO), and ZnO are currently used for their antimicrobial activity [69]. Adherence of microorganisms to a substrate, enables the microbes to evade the normal flushing action of saliva and allows the microbes to survive harsh growth conditions [70].

Kishen *et al.* demonstrated that the quantum size effect of nanoparticles permits them to exhibit superior interaction with bacteria and dentin substrate [71]. They further revealed that when cationic nanoparticles in an aqueous suspension were allowed to settle onto the dentin surface with negative charge, the cationic nanoparticles adhere to the dentin surface via an electrostatic interaction. They also demonstrated that although the interaction between nanoparticles and dentin was weak and easily disrupted, it could impede bacterial re-colonization and biofilm formation .

Chitosan (CS) is a natural non-toxic biopolymer derived from the deacetylation of chitin. It binds to negatively charged surfaces and has excellent antimicrobial and antifungal activities [72]. The exact mechanisms of the antibacterial action of CS and its derivatives have still not been elucidated. Nonetheless, Rabea *et al.* stated that the electrostatic interaction between the positively charged CS nanoparticles and the negatively charged bacterial cell membrane is believed to alter bacterial cell permeability and loss of function [72]. Kishen *et al.* examined the antimicrobial properties of ZnO and resin-based root canal sealers loaded with CS and ZnO nanoparticles [71]. Findings demonstrated that the addition of antibacterial nanoparticles in root canal sealers improves the *direct* and *diffusible* antibacterial effects in root canal sealers based on a direct antibacterial assay and a membrane-restricted antibacterial assay, respectively.

In another part of their study, Kishen *et al.* indicated that treatment of root dentin with ZnO nanoparticles, ZnO–CS mixed nanoparticles, CS-layer-ZnO nanoparticles, or CS nanoparticles, produced an 80–95% reduction in the adherence of *E. faecalis* to dentin [73]. They further revealed that root dentin treated with CHX and then with nanoparticles, shows the maximum reduction (97%) in bacterial adherence [73].

Shrestha *et al.* assessed the efficacy of CS nanoparticles and ZnO nanoparticles in eliminating bacterial biofilm and the effect of aging (conditioning with tissue fluids) on their antibacterial properties [74]. *E. faecalis* strains in planktonic and biofilm forms were tested in this study. It was demonstrated that the rate of bacterial killing by nanoparticles depended on the concentration and duration of interaction. Total elimination of planktonic bacteria was observed in contrast to the biofilm bacteria, which survived even after 72 h of interaction. Both CS nanoparticles and ZnO nanoparticles were found to retain their antibacterial properties after aging for 90 days.

***Bioactive glass***

Bioactive glass (BAG) consists of SiO_2_, Na_2_O, CaO_2_, and P_2_O_5_ at different concentrations [75]. It has received considerable interest in root canal disinfection due to antibacterial properties. Stoor *et al.* attributed the antibacterial mechanism of BAG to its high pH, osmotic effects and Ca/P precipitation [76]. Zehnder *et al.* demonstrated that compared CH, BAG showed significantly less antibacterial effects as an intracanal medicament [77]. In addition, Gubler *et al.* showed that BAG did not effectively prevent recontamination of instrumented root canals [78].

Incorporation of nanometric BAG fillers into polyisoprene (PI) and polycaprolactone (PCL) root filling materials, rendered the resulting composite material bioactive and permitted improved mineralization [79].

***Laser***

Seal *et al.* compared the bacterial killing of *Streptococcus intermedius* biofilms in root canals using lethal photosensitization with various combinations of photosensitizer concentration and laser light dose or 3% NaOCl irrigation [80]. Findings showed that the combined use of a photosensitizing agent and a low power laser directed at the access cavity was bactericidal to *S. intermedius* biofilms in root canals but was unable to achieve total kill, unlike 3% NaOCl. Araki *et al.* evaluated the effect of Er: YAG laser on the apical third of the roots of newly extracted teeth to eliminate microbial contamination on root apex surface and found that it may be considered as an effective tool for removal of apical biofilm [81].

In an *in vitro *study, Bergmans *et al.* found that endodontic pathogens that grew as biofilms were difficult to eradicate even upon direct laser exposure [82]. Soukos *et al.* investigated the effects of photodynamic therapy (PDT) on endodontic pathogens in planktonic phase as well as on *E. faecalis* biofilms in experimentally infected root canals of extracted teeth [83]. Strains of microorganisms were sensitized with methylene blue (25 µg/mL) for 5 min followed by exposure to red light of 665 nm with an energy fluence of 30 J/cm. Methylene blue fully eliminated all bacterial species except for *E. faecalis* (53% killing). The same concentration of methylene blue in combination with red light (222 J/cm) was able to eliminate 97% of *E. faecalis* biofilm bacteria in root canals using an optical fiber with multiple cylindrical diffusers that uniformly distributed light at 360 degrees. Noiri *et al.* examined the *in vitro* effect of Er: YAG laser against biofilms made of *Actinomyces naeslundii, E. faecalis, Lactobacillus casei, Propionibacterium acnes, Fusobacterium nucleatum, Porphyromonas gingivalis*, or *Prevotella nigrescens*. Findings demonstrated that the Er: YAG laser was effective against biofilms of 6 of the bacterial species examined, except for those formed by *L. casei*. After irradiation, the numbers of viable cells in the biofilms significantly decreased, whereas atrophic changes in bacterial cells and reductions in biofilm cell density were seen morphologically. They concluded that Er: YAG lasers might be suitable for clinical application as a suppressive and removal device of biofilms in endodontic treatments [26].

On the whole, although most studies support the efficacy of lasers against endodontic biofilms, further studies should be conducted to confirm this.

***Ozone***

Ozone (O_3_) is an energized, unstable gaseous form of oxygen that readily dissociates back into oxygen (O_2_), liberating a reactive form of oxygen, aka the singlet oxygen (O_1_). The singlet oxygen is capable of oxidizing cells. It has been suggested that ozone accomplishes its antimicrobial efficacy without developing drug resistance [84]. Ozone gas (HealOzone; KaVo Dental, Biberach, Germany) is currently used clinically for endodontic treatment. However, results of studies on its efficacy against endodontic pathogens have been inconsistent. This inconsistency is attributed to the lack of information about the optimum duration of application and concentration that should be used [85]. In order to achieve a concentration that is relatively non-toxic toward periapical and oral mucosal tissues, the ozone gas concentration currently used in Endodontics is 4 g/m^3^. This concentration has been shown to be slightly less cytotoxic than 2.5% NaOCl [85].

Aqueous ozone (up to 20 mg/mL) showed essentially no toxicity to oral cells *in vitro* [85]. Hems *et al.* showed that ozone had an antibacterial effect on planktonic E. faecalis cells and those suspended in fluid, but little effect on cells embedded in a biofilm structure [86]. Furthermore, the antibacterial efficacy of ozone was not comparable with that of NaOCl. Huth *et al.* assessed the antimicrobial efficacy of aqueous (1.25–20 mg/mL) and gaseous ozone (1–53 g/m^3^) as an alternative antiseptic against endodontic pathogens in suspension and in a biofilm model [87]. *E. faecalis, Candida albicans, Peptostreptococcus micros, *and* Pseudomonas aeruginosa* were grown in planktonic culture or in mono-species biofilms in root canals for 3 weeks. It was concluded that highly concentrated gaseous and aqueous ozone was dose-, strain-, and time-dependently effective against the tested microorganisms in suspension and in the biofilm test model.

Viera *et al.* assessed the antimicrobial efficacy of dissolved ozone against planktonic and biofilm models of *Pseudomonas fluorescens *[88]. Findings showed that even low concentration of ozone (0.1–0.3 ppm) was able to completely kill bacteria after 15 or 30 min of contact time. However, the disinfectant action of ozone on biofilm models was less effective compared with planktonic bacteria. In the biofilm models, only a decrease of two orders of magnitude was achieved. No increase in the anti-biofilm efficacy was observed with increases in contact time.

Kustarci *et al.* evaluated the antimicrobial activity of a potassium titanyl phosphate (KTP) laser and gaseous ozone in experimentally infected root canals [89]. It was found that both the KTP laser and gaseous ozone have a significant antibacterial effect on infected root canals, with the gaseous ozone being more effective than the KTP laser. However, 2.5% NaOCl was superior in its antimicrobial abilities compared with the KTP laser and gaseous ozone. Silveira *et al.* has claimed that ozone dissolved in oil can be used as an intracanal medicament [90].

## Conclusion

According to the latest data, removal of the smear layer is an essential of root canal disinfection and sealing. Contrary to the vulnerable planktonic state, bacteria are protected from the antibacterial agents in biofilms. To date, many methods and antibacterial agents have been proposed against biofilms that are effective within a wide range of activity.
